# Rare Case of Giant Asymptomatic Left Coronary Artery Aneurysm of 10 cm Associated with Coronary Cameral Fistula

**DOI:** 10.7759/cureus.3566

**Published:** 2018-11-09

**Authors:** Ali Afzal, Syed Mobin, Mohamad Sharbatji, Hussain Nawaz, Muqeet Siddiqui

**Affiliations:** 1 Internal Medicine, Florida Hospital, Orlando, USA; 2 Internal Medicine, University of Central Florida College of Medicine, Orlando, USA; 3 Internal Medicine, Aga Khan University Hospital, Lahore, PAK; 4 Family Medicine, Florida Hospital, Orlando, USA

**Keywords:** coronary artery fistula, giant coronary artery aneurysm, 10cm

## Abstract

A giant coronary artery aneurysm (CAA) associated with a coronary cameral fistula is an extremely rare finding. Most cases of CAAs are asymptomatic. The incidence of CAA varies between 0.3% to 5.3%. Due to advancements in diagnostic technologies, the incidence of CAA is on the rise. Even in the modern days of medical science, the clinical course of CAA is still unpredictable and the suitable timing for the treatment of CAA is still open to debate. We reported a case of a giant coronary artery aneurysm in a 38-year-old female, which was 9.4 x 9.7 x 9.2cm in size, arising from the left coronary artery, extending into the proximal circumflex, and draining into the right atrium, forming a fistula tract. The patient was diagnosed with the help of coronary computed tomography (CT) and cardiac catheterization after which surgery was performed to repair the aneurysm and fistula. Postoperatively, the patient recovered without any complication.

## Introduction

A coronary artery aneurysm (CAA) is a rare anomaly that was first discovered in 1761 on an autopsied patient [1]. A coronary artery aneurysm is defined as localized ectasia of the lumen of the coronary artery by 1.5 times of its normal adjacent segments. A CAA is most commonly seen in the right coronary artery (RCA) followed by the left anterior descending (LAD) and left circumflex [2-3]. A giant CAA is defined as an aneurysm of the coronary artery that is > 4 cm in diameter [4]. The prevalence of a giant CAA is only 0.02% to 0.2%, which is even lower than the prevalence of other small CAAs [5]. There is a small number of cases in which giant CCAs are associated with fistulas that drain into cardiac chambers like the right atrium, right ventricle, or pulmonary artery [6]. Such a type of anomaly in which the coronary artery forms an abnormal connection with the cardiac chambers is called a coronary cameral communication/fistula. Small coronary cameral fistulas are asymptomatic and are incidentally found on a coronary angiogram while larger coronary cameral fistulas may present with the symptoms of ischemia mostly because of coronary steal, dyspnea, or sudden death. Giant coronary aneurysms associated with coronary cameral fistulas need to be treated as soon as diagnosed because of an increased risk of rupture or myocardial ischemia [7]. Because of the rarity of giant CAAs and coronary cameral fistulas, there is no gold standard or evidence-based treatment for this condition but surgery is the mainstay treatment for symptomatic giant CAAs.

## Case presentation

**Figure 1:**
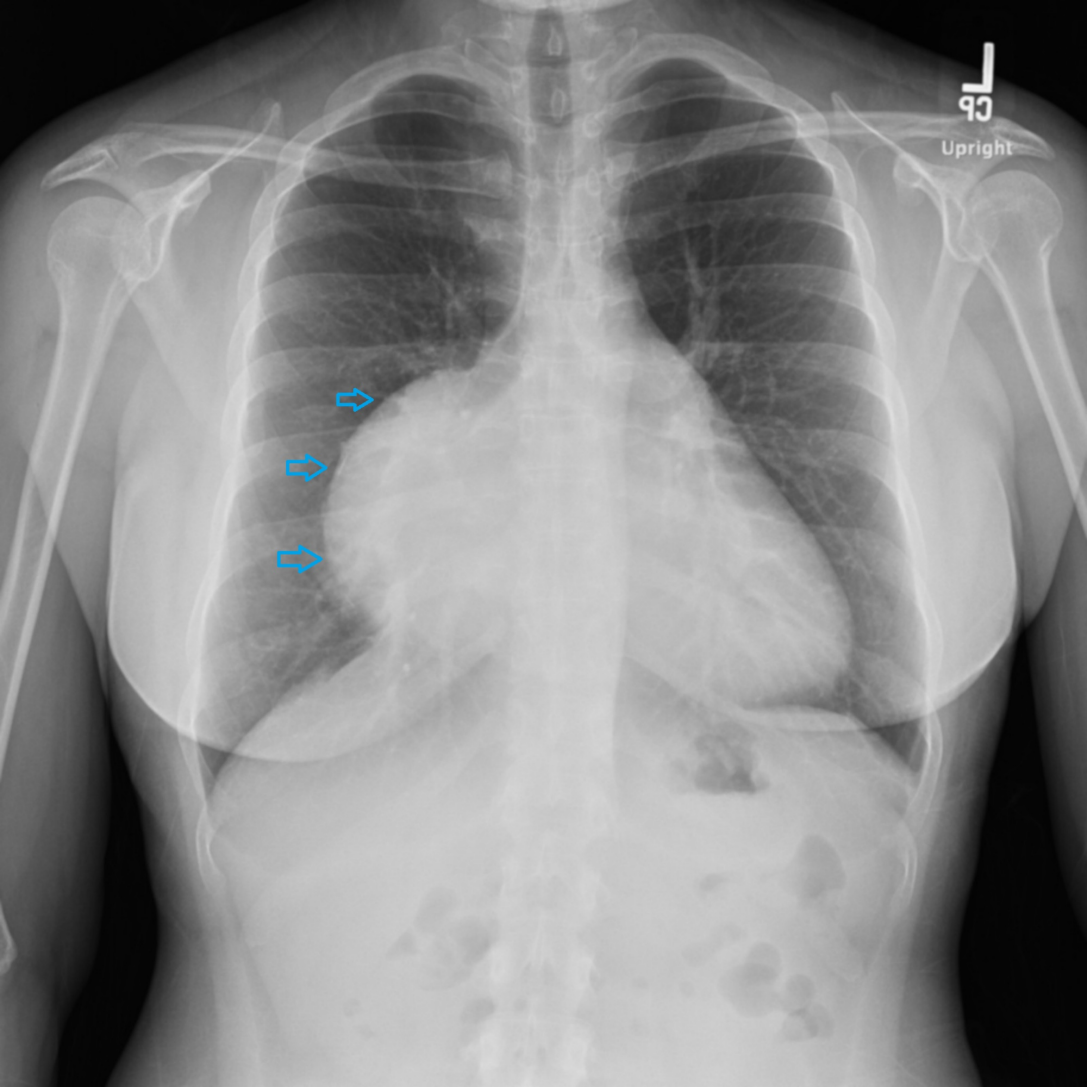
A large mass-like opacity associated with a cardiac silhouette on the right side

A 38-year-old female with no past medical history came to the hospital with complaints of episodic chest discomfort, mild dyspnea, and occasional non-productive cough. On physical examination, she was hemodynamically stable without any pathological finding. A chest x-ray was done and it showed mass-like opacities abutting the right heart (Figure 1).

**Figure 2:**
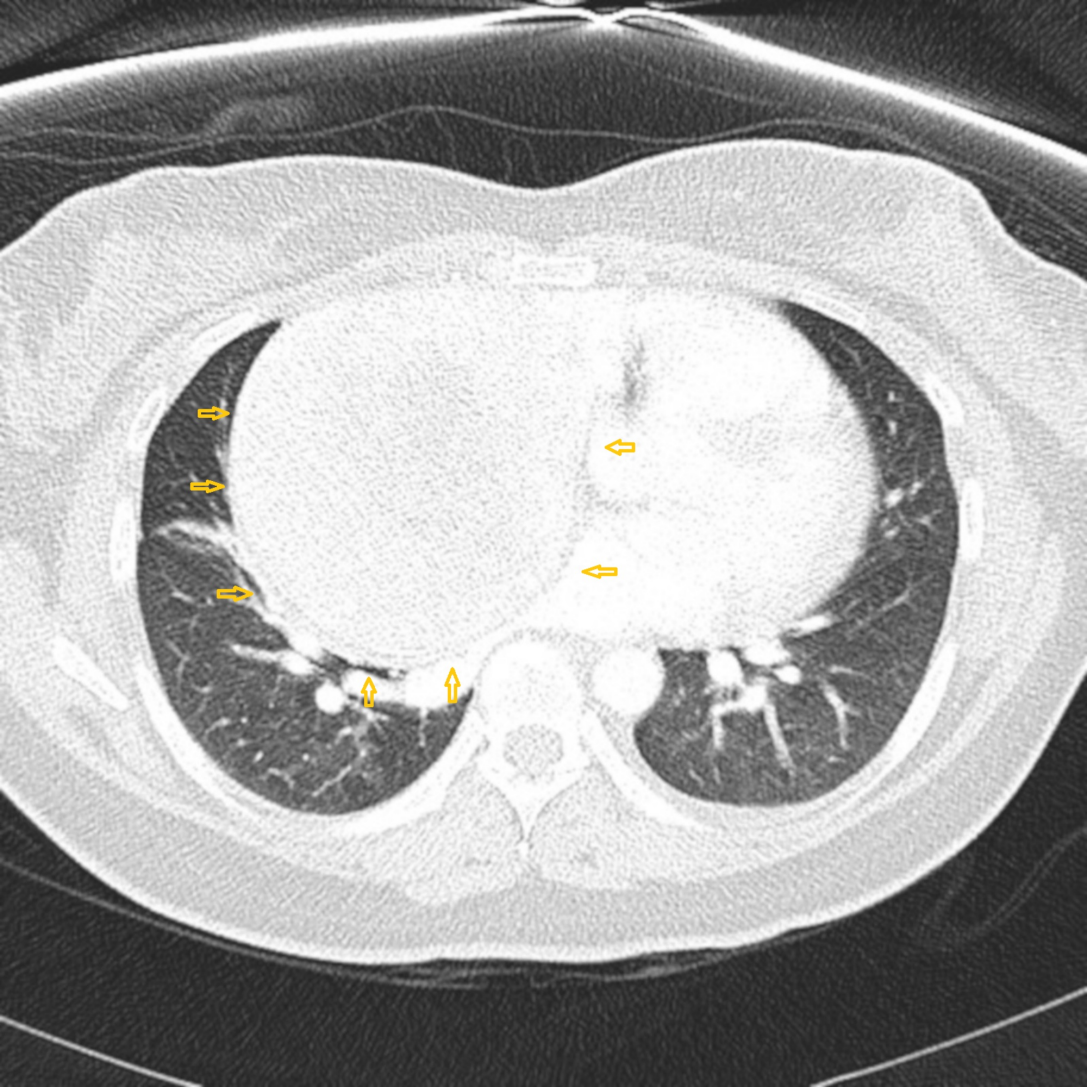
CT chest with contrast showing a large, well-circumscribed, heterogeneously enhancing mass showing peripheral calcification in the right mediastinum CT: computed tomography

To have a better idea of the cause of opacity, a further assessment with computed tomography (CT) chest with contrast was ordered and it showed a large, well-circumscribed, heterogeneously enhancing mass of 10 cm with peripheral calcification in the right mediastinum. Also, there was a dilated vessel along the posteromedial and inferior of the mass (Figure 2).

**Figure 3:**
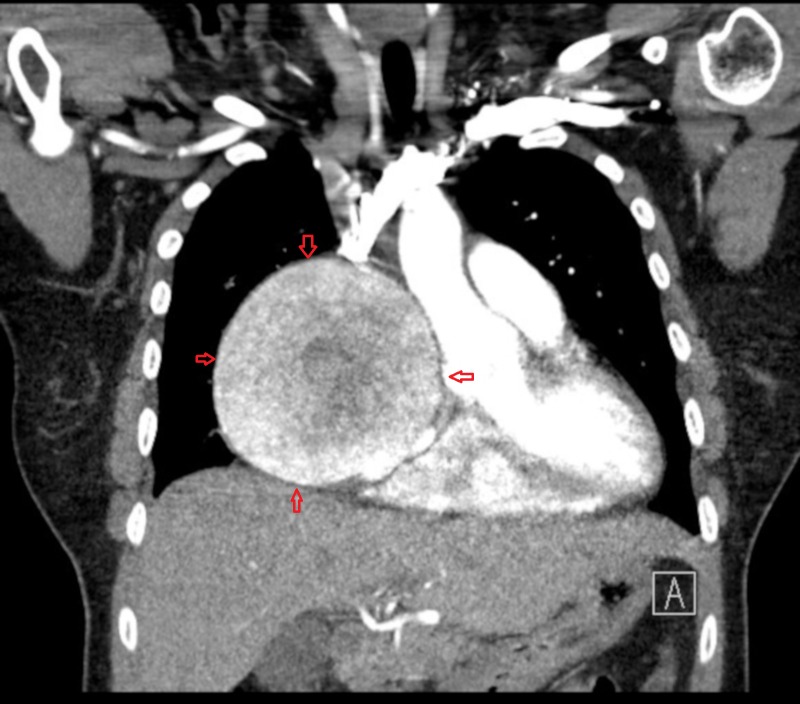
CCTA showing an anomalous vessel arising from the proximal left circumflex artery and giving rise to a 9.7 cm coronary artery aneurysm CCTA: coronary computed tomography angiography

**Figure 4:**
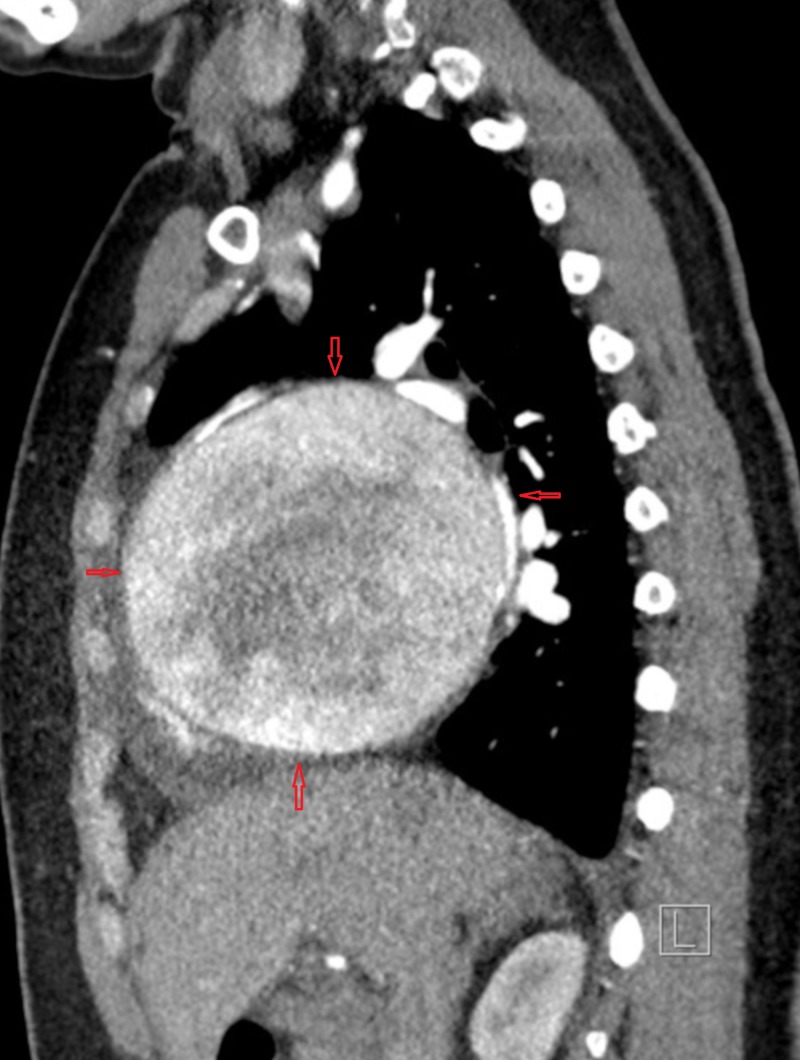
CT coronary showing a giant coronary artery aneurysm CT: computed tomography

After the chest CT findings, the patient was admitted to the hospital for further evaluation and cardiac consult was called. In order to differentiate if the mass was due to some tumor or some anomaly of the coronary vessel, coronary CT angiography with contrast was ordered. A subsequent coronary CT angiography (CCTA) showed a 9.7 cm aneurysm and an anomalous vessel emanating from the left coronary artery and the proximal circumflex, fistulizing into the right atrial appendage. CCTA also showed a dilated right atrium likely due to fistula formation (Figures 3-4).

When the right heart catheterization was performed, a rise in saturation was noted in the right side of the heart due to shunting of blood from the left side to the right side of the heart as a result of coronary cameral fistula formation between the right heart and the anomalous vessel originating from the left coronary artery and proximal circumflex artery. On transesophageal echocardiogram (TEE), an enlarged right atrial chamber was noted due to the fistulous tract draining into the right atrium. The cardiothoracic department then scheduled the patient for surgery. Sternotomy was performed to repair the coronary artery aneurysm with ligation and resection of the coronary cameral fistula and repair of the right atrium. Surgery went well and no intra-operative and postoperative complications were noted.

## Discussion

Giant coronary artery aneurysm (CAA) is an extremely rare anomaly of coronary vessels affecting only a small percentage of the population. The prevalence of giant CCAs associated with coronary cameral fistulas is low and varies between 0.03% and 0.3%. The odds to report a case of giant CAA that is > 5 cm are extremely rare. The most commonly involved vessel, where coronary cameral fistulas originate, is right coronary artery (RCA) (55%) followed by the left coronary artery (LCA) (35%) while the major draining sites of the fistula are the right ventricle (40%), right atrium (26%), pulmonary artery (11%), and, less commonly, the superior vena cava or coronary sinus [8]. In our case report, the patient's coronary cameral fistula originated from the LCA and proximal left circumflex artery (LCX) with drainage into the right atrium, which is an unusual site for fistula origin and drainage. Also, CAAs are more frequently seen in males over the age of 60 but our patient of CAA was a 38-year-old female.

The pathological mechanism of CAA is still controversial. It could be due to acquired or inherited etiology. The most common cause of CAA in adults is atherosclerosis; other causes include congenital heart disease, trauma, Ehlers-Danlos syndrome, Kawasaki disease, Marfan syndrome, Takayasu arteritis, polyarteritis nodosa, syphilitic aortitis, scleroderma, and systemic lupus erythematosus [9]. Most cases of CAAs are asymptomatic. However, patients with a giant CAA may have symptoms of chest pain, dyspnea, or palpitation. Patients of giant CAAs who have an associated coronary cameral fistula also present with symptoms of angina due to the shunting of blood [10]. Our patient presented with the symptoms of episodic chest pain, mild dyspnea, and occasional cough.

There are a variety of imaging studies that can diagnose a coronary cameral fistula but coronary angiography is the mainstay diagnostic test. It is rare for a CAA to be seen on a chest X-ray. Only when the CAA size is extraordinarily large can it be seen on chest X-ray (CXR). Other imaging studies used to diagnose CAA are echocardiography, CT, and magnetic resonance imaging (MRI) [11]. Owing to the rarity and unavailability of controlled trials, no evidence-based guidelines exist for the optimal management of giant coronary artery aneurysms. A number of studies recommend that all patients with a coronary artery fistula be considered for surgical correction, especially in cases of giant aneurysms and the progressive enlargement of the coronary cameral fistula [12]. One of the main indications for the surgical correction of a coronary cameral fistula is increased shunting of blood from the left to the right side of the heart, as seen in our patient. Although simple observation is sufficient for small asymptomatic aneurysms, it is not sufficient for a giant CAA with an associated fistula﻿.

## Conclusions

This case report demonstrates a rare and unique finding of a giant coronary artery aneurysm of almost 10 cm arising from the left coronary artery and proximal circumflex artery associated with a coronary cameral fistula in an otherwise young, healthy female. Surgical referral and management may be crucial in preventing significant morbidity and mortality of patients with giant CAAs.
